# Epitope Mapping of Avian Influenza M2e Protein: Different Species Recognise Various Epitopes

**DOI:** 10.1371/journal.pone.0156418

**Published:** 2016-06-30

**Authors:** Noor Haliza Hasan, Esmaeil Ebrahimie, Jagoda Ignjatovic, Simson Tarigan, Anne Peaston, Farhid Hemmatzadeh

**Affiliations:** 1 School of Animal and Veterinary Sciences, The University of Adelaide, SA, Australia; 2 Institute for Tropical Biology and Conservation, University Malaysia Sabah, Sabah, Malaysia; 3 Australian Centre for Antimicrobial Resistance Ecology, School of Animal and Veterinary Sciences, The University of Adelaide, SA, Australia; 4 School of Information Technology and Mathematical Sciences, Division of Information Technology, Engineering and the Environment, University of South Australia, Adelaide, Australia; 5 School of Biological Sciences, Faculty of Science and Engineering, Flinders University, Adelaide, Australia; 6 School of Veterinary and Agricultural Sciences, The University of Melbourne, Vic, Australia; 7 Indonesian Research Center for Veterinary Sciences, Bogor, Indonesia; Georgia State University, UNITED STATES

## Abstract

A common approach for developing diagnostic tests for influenza virus detection is the use of mouse or rabbit monoclonal and/or polyclonal antibodies against a target antigen of the virus. However, comparative mapping of the target antigen using antibodies from different animal sources has not been evaluated before. This is important because identification of antigenic determinants of the target antigen in different species plays a central role to ensure the efficiency of a diagnostic test, such as competitive ELISA or immunohistochemistry-based tests. Interest in the matrix 2 ectodomain (M2e) protein of avian influenza virus (AIV) as a candidate for a universal vaccine and also as a marker for detection of virus infection in vaccinated animals (DIVA) is the rationale for the selection of this protein for comparative mapping evaluation. This study aimed to map the epitopes of the M2e protein of avian influenza virus H5N1 using chicken, mouse and rabbit monoclonal or monospecific antibodies. Our findings revealed that rabbit antibodies (rAbs) recognized epitope ^6^EVETPTRN^13^ of the M2e, located at the N-terminal of the protein, while mouse (mAb) and chicken antibodies (cAbs) recognized epitope ^10^PTRNEWECK^18^, located at the centre region of the protein. The findings highlighted the difference between the M2e antigenic determinants recognized by different species that emphasized the importance of comparative mapping of antibody reactivity from different animals to the same antigen, especially in the case of multi-host infectious agents such as influenza. The findings are of importance for antigenic mapping, as well as diagnostic test and vaccine development.

## Introduction

Matrix protein 2 (M2) of avian influenza virus (AIV) is a 97 amino acids (aa) protein encoded by RNA segment 7 of the influenza A virus (IAV) [1]. It is translated from spliced mRNA and shares a common start codon with the matrix 1 (M1) protein and the first nine aa, while the remaining 88 aa continues at the second (+1) open reading frame [1, 2]. In its native state, M2 is a homotetrameric type III integral membrane protein composed of three domains; namely, a 54 aa cytoplasmic domain located in the viral envelope or cytoplasmic membrane of infected cells, a 19 aa transmembrane domain, and an N-terminal 24 aa ectodomain (M2e) which is exposed on the surface of the virus infected cells and on the viral particles [1, 3–5]. In the infected cell the M2 protein forms an ion channel which is vital for viral genome delivery into the host cell during virus entry [2, 3, 5–8]. Briefly, M2 ion channel activity is activated by acidification of virus-containing endosomes after internalization of the virus particle into the host cell via clathrin-dependant and –independent mechanisms [9, 10].

Amino acids 1–9 of the M2e protein are highly conserved across AIV strains, while minimal aa variation is observed for residues 10 to 24, making it an attractive target for AIV universal vaccine development [2, 11–20]. The M2e protein is low in copy number on the virus particle, but it is abundantly expressed on the surface of an infected cells [3, 21]. This differential epitope density between infected cells (high) and a mature virion (low) [15, 22] is the key feature for its recommendation as a marker for differentiating infected animals in vaccinated population (DIVA), a strategy used in AIV surveillance [23, 24].

The sensitivity and specificity of M2e-based DIVA have been demonstrated in our previous works [25–27]. This raised our interest towards the potential use of M2e in a competitive enzyme-linked immunosorbent assay (ELISA) format as a surveillance tool for AIV infection. The principle of competitive ELISA lies in the ability of the test subject antibody (e.g. chicken) to inhibit competitor antibodies, usually produced in rabbit or mouse, from binding to the target antigen. Hence, it is important for the competitor antibodies to react with the same viral epitopes as the antibodies produced by the test species. Such an ELISA format has been successfully demonstrated for the nucleoprotein of AIV, which has been proven to be reliable and applicable for multispecies surveillance [28–30]. However, M2e-based competitive ELISA is a better alternative DIVA test for an AIV surveillance tool, especially in the highly pathogenic AIV H5N1 endemic countries, where poultry vaccination using inactivated AIV is practiced.

It is accepted that due to differences in the germline gene repertoire in different species, accompanied by distinct mechanisms for generation and affinity maturation of antibodies, antigenic determinants recognized by a host can vary from one species to another [31–33]. Earlier studies on M2e protein for vaccine development have reported several antigenic determinants identified by anti-M2e antibodies isolated from rabbit, mouse and human [20, 34, 35]. In most cases, the M2e epitopes recognized were located in the region that span from the N-terminal to the middle region of M2e, and vary in length from 5 residues (^2^SLLTE^6^) [35], up to 15 residues ^2^SLLTEVETPIRNEWG^16^ [20, 34]. Here, we describe epitope mapping using anti-M2e antibodies from chicken, mouse and rabbit to identify the M2e antigenic determinants for each antibody group, and to assess the most suitable animal source of anti-M2e antibodies in M2e-based competitive ELISA as an advanced DIVA test for H5N1 infections in poultry.

## Material & Methods

### Peptides for mouse and rabbit immunization and antigenic mapping

Peptide immunization for mouse and rabbit was done using the 17 amino acid (aa) M2e peptide (M2e_2-18_), corresponding to residues 2 to 18 of HPAIV H5N1 Indonesian strain A/Chicken/West Java/PWT-WIJ/2006, (^2^SLLTEVETPTRNEWECK^18^) [25–27]. It was conjugated with Keyhole Limpet Hemocyanin (M2e-KLH) at the C-terminal end for the anti-M2e antibodies production in mice (Abmart, Shanghai, China) and rabbits (Peptide 2, Chantilly, Virginia, USA).

M2e-mapping was done using two sets of overlapping short peptides spanning M2e_2-24_. Set 1 included eight peptides of 9–10 aa length (WatsonBio, Houston, Texas) with two aa offsets each; while set 2 included three peptides of 14 aa length (Abmart, Shanghai, China) with three aa offsets each (Table 1). M2e_2-18_ was used for anti-M2e antibodies screening in indirect ELISA, as well as the positive antigen control in mapping ELISA, instead of M2e_2-24_, as both showed similar reactivity in previous study [26]. All peptides used were of >90% purity as determined by high performance liquid chromatography analyses.

**Table 1:** Overlapping peptides covering the full length H5N1 M2e protein (M2e_2-24_), designed with 10 amino acid (aa) with 2 offsets, and 14 aa with 3 offsets each. Peptide M2e_2-18_ was used as a control antigen in place of M2e_2-24_.

Peptide designation	Peptide sequence	Peptide length
M2e _2–11_	^2^SLLTEVETPT^11^	9–10 aa
M2e _4–13_	^4^LTEVETPTRN^13^	
M2e _6–15_	^6^EVETPTRNEW^15^	
M2e _8–17_	^8^ETPTRNEWEC^17^	
M2e _10–19_	^10^PTRNEWECKC^19^	
M2e _12–21_	^12^RNEWECKCSD^21^	
M2e _14–23_	^14^EWECKCSDSS^23^	
M2e _16–24_	^16^ECKCSDSSD^24^	
M2e _5–18_	^5^TEVETPTRNEWECK^18^	14 aa
M2e _8–21_	^8^ETPTRNEWECKCSD^21^	
M2e _11–24_	^11^TRNEWECKCSDSSD^24^	
M2e _2–18_	^2^SLLTEVETPTRNEWECK^18^	17 aa

### Antibodies (sera)

Three different sources of anti-M2e antibodies were used in this study, namely chicken polyclonal antibodies (cAbs), mouse monoclonal antibodies (mAbs), and rabbit polyclonal antibodies (rAbs) (Table 2). CAbs were produced as described previously [25, 27]. In brief, commercial layer chicks were inoculated with inactivated H5N1 AI vaccine (Medivac-AI, PT Medion, Bandung, Indonesia), once (16 weeks of age), twice (12 and 16 weeks of age) or three times (8, 12 and 16 weeks of age). All chicks were challenged with live H5N1 strain (either A/Ck/West Java/PWT-WIJ/2006, or A/Ck/West Java/Sbg-29/2007) two weeks after the last vaccination. All challenge experiments were conducted in the Biosecurity level 3 (BSL3) facilities at the Indonesian Research Centre for Veterinary Science, Bogor, Indonesia. Collected sera were tested for M2e reactivity using indirect M2e ELISA [25, 26]. Reference H5N1 sera (A/Chicken/Scotland/59) was obtained from the Veterinary Laboratory Agency (New Haw, Addlestone, UK) as described previously [26].

**Table 2:** Antibody types and animals used for the generation of antibodies either by H5N1 virus challenge, or KLH-M2e_2-19_ peptide immunization.

Antibody type	Antibody designation	Immunogen
Chicken polyclonal antibodies	PL64	A/Ck/West Java/PWT-WIJ/2006
	PL80	
	2D10	A/Ck/West Java/Sbg-29/2007: MSLLTEVETPTRNEWECKCIDSSD
	2B2	
	2B47	
	2A17	
	Reference H5N1 sera	A/Chicken/Scotland/59
Mouse monoclonal antibodies	1N5	M2e_2-19_ peptide: SLLTEVETPTRNEWECKC-KLH
	2D16	
	2E14	
	2G14	
	3D23	
	3H4	
Rabbit polyclonal antibodies	rAb-1	
	rAb-2	
	rAb-3	
	rAb-4	
	rAb-5	
	rAb-6	
	rAb-7	
	rAb-8	

Hybridoma cells producing anti-M2e mAbs were produced by Abmart (Shanghai, China) following immunization of mice with M2e_(2–19)_-KLH peptide. Briefly, six female BALB/c mice were injected subcutaneously at multiple sites with an emulsion contained 0.05 mg KLH-M2e peptide mixed with complete Freund’s adjuvant (CFA). Immunization was done four times 14 days apart. Booster injections were given 14 days after last immunization with 0.05 mg KLH-M2e peptide in incomplete Freund’s adjuvant (IFA). Serum sampling was done seven days after the third and fourth immunization and sera tested for anti-M2e antibodies using indirect M2e-ELISA. Fusion of myeloma cells and spleen cells was followed by another indirect M2e-ELISA screening. Selected clones of hybridoma cells were expanded and grown in Dulbecco’s modified Eagles medium (DMEM) high glucose with L-glutamine (HyClone, GE Healthcare) with 15% foetal bovine serum (HyClone, GE Healthcare) and 1% (v/v) penicillin and streptomycin (Gibco, Thermofisher Scientific). MAb supernatants from cell culture were column purified using Pierce Recombinant Protein A Agarose (Thermofisher Scientific). No significant difference was observed between the column purified and precipitated mAb in indirect ELISA. Thus, for the experiments described here, the mAb supernatants were precipitated using 50% saturated solution of ammonium sulphate and the protein pelleted was resuspended in sterile phosphate saline buffer (PBS) and stored at -20°C until required.

Eight New Zealand White rabbits with the average age of 6 months were chosen to obtain hyperimmune serum against the M2e peptide. Rabbits were inoculated at five subcutaneous sites with an emulsion that contained 0.1 mg of KLH-M2e peptide mixed with CFA. The rabbits received booster injections containing 0.1 mg KLH-M2e peptide in IFA at day 14 and 28. Blood was collected two weeks after the final immunization and antisera tested using indirect M2e-ELISA.

### Indirect M2e-ELISA and antigenic mapping

All peptides were dissolved in diethylpyrocarbonate (DEPC)-treated water (Bioline) to a final concentration of 1 mg/ml. Peptides were diluted with 0.1 M carbonate-bicarbonate buffer, pH 9.6 (0.1 M Na_2_CO_3_, 0.1 M NaHCO_3_) to the final concentration of 10 μg/ml, and 100 μl was added to each well of a 96-well flat bottom microtiter plate (Maxisorp, NUNC) and incubated at 4°C overnight. The coated plates were washed five times with PBS containing 0.05% Tween 20 (PBS-T) and blocked with 2% BSA in PBS (200 μl/ well) for 2 hrs at room temperature (RT). The chicken test sera were diluted with the high salt dilution buffer (HS-DB: 0.1 M Tris pH 7.4, 0.5 M NaCl, 1 mM Na_2_EDTA, 2% w/v BSA, 3% w/v Triton X-100, 3% w/v Tween 20) [25, 26], and mouse and rabbit sera were diluted in PBS containing 1% BSA and 0.05% Tween 20 (PBS-BSA-T) with the dilution of 1:100 for all sera. The blocked plates were washed for five times with PBS-T before the diluted serum was added into wells containing each peptide (100 μl/well). After 1 hr of incubation at RT, the plates were subjected to another five rounds of washing. Species-specific antibodies conjugated with horseradish peroxidase (HRP) enzymes were prepared by dilution of anti-chicken HRP with HS-DB, and anti-mouse HRP (Sigma) and anti-rabbit HRP were diluted with PBS-BSA-T. Diluted secondary antibodies were added to each well (100 μl/well), followed by 1 hr incubation at RT. After washing, the substrate solution [100 μg/ml of tetramethylbenzidine substrate (TMB) (Sigma, St Louis, MO, USA)] in citrate buffer (pH 8) containing hydrogen peroxide (100 μl of 0.6% H_2_O_2_) was added (100 μl/ well) and incubated at RT for 5–20 minutes before the reaction development was stopped with stop buffer (1 M sulphuric acid) (50 μl/ well). The optical density (OD) of each well was determined at OD 450 nm using the BioRad Benchmark Plus Microplate Reader (BioRad, Hercules, USA).

### Statistical and bioinformatics analyses

Each antigenic peptide was tested in three dilutions with two replicas each. A range of univariate and multivariate analyses were employed in this study as previously described [36], using MINITAB 17 statistical package [37]‎. The mean OD_450_ values for the antigen negative wells were subtracted from the mean OD_450_ values of antigen positive wells to get the corrected OD_450_ values. One-way ANOVA and pair-wise mean comparison by Tukey test was used to compare the corrected ELISA values of different antigenic peptides within each type of antibody (chicken, mouse, and rabbit). Antibody reactivity to the M2e peptides was considered as strong (>1.00), medium (0.50–1.00), weak (0.25–0.50) and negative (<0.20) in reference to its OD_450_ value.

Clustering based on Average Linkage algorithm was used to illustrate the similarities/differences between different peptides in reaction with each type of antibody. The same method was used to cluster antibodies against antigenic peptides. Hydrophobicity plot of M2e protein (aa 2–24) was constructed using the BioEdit software (North Carolina State University) and CLC Genomics (QIAGEN) [38].

### Ethics statement

Animal work carried out at the Indonesian Research Centre for Veterinary Science, Bogor, Indonesia was approved by the Research Committee of Indonesian Research Centre for Veterinary Science. The experimental chickens were handled by an expert veterinarian in animal studies based on the guidelines of the National Health and Medical Research Council of Australia. The animals were checked daily for clinical signs, morbidity, and mortality. All chickens were bled via brachial vein and by cardiac puncture at the terminal step just after CO_2_ euthanasia. humane endpoint was not applied in this study.

## Results

### Chicken, mouse and rabbit antibodies selection using indirect-M2e ELISA

Positive anti-M2e cAbs were selected based on findings from previous reports [25, 26], where end-point HI antibody titers for all cAbs were approximately 1:512 dilutions (data not shown). Meanwhile, positive anti-M2e mAbs and rAbs showed ELISA titers between 1:1600 to 1:3200, and 1:800 to 1:1600, respectively. As expected, comparison of mean OD_450_ readings for chicken, mouse and most rabbit antibodies showed strong (OD_450_ >1.0) reactivity to the M2e_2-18_ (Table 3). All results for statistical analysis can be found in S1 File.

**Table 3:** Mean OD_450_ readings for chicken (^a^^,^ ^b^^,^ ^c^), mouse (^d^) and rabbit (^e^) antibodies reactivity to the M2e peptide.

Antibody	OD450 on Peptide																							
	M2e _2–18_		M2e_11-24_		M2e_8-21_		M2e_5-18_		M2e_16-24_		M2e_14-23_		M2e_12-21_		M2e_10-19_		M2e_8-17_		M2e_6-15_		M2e_4-13_		M2e_2-11_	
**2A17**^a^	2.11	✓✓✓	0.02	-	0.92	✓✓	1.66	✓✓✓	0.04	-	0.05	-	0.04	-	0.81	✓✓	0.87	✓✓	0.17	-	0.03	-	0.06	-
**2B2**^a^	2.07	✓✓✓	0.01	-	0.58	✓✓	1.51	✓✓✓	0.02	-	0.04	-	0.05	-	0.26	✓	0.35	✓	0.20	-	-0.01	-	-0.01	-
**2B47**^a^	1.85	✓✓✓	-0.22	-	1.20	✓✓✓	1.32	✓✓✓	-0.08	-	-0.27	-	-0.12	-	-0.20	-	1.13	✓✓✓	-0.11	-	-0.21	-	-0.13	-
**2D10**^a^	2.33	✓✓✓	0.04	-	2.14	✓✓✓	2.14	✓✓✓	0.10	-	0.10	-	0.14	-	2.02	✓✓✓	2.24	✓✓✓	0.17	-	0.09	-	0.05	-
**PL64**^b^	2.29	✓✓✓	0.04	-	0.01	-	0.76	✓✓	0.08	-	0.08	-	0.14	-	0.02	-	0.10	-	0.10	-	0.07	-	0.10	-
**PL80**^b^	2.34	✓✓✓	0.10	-	-0.05	-	1.13	✓✓✓	0.16	-	0.02	-	0.07	-	-0.02	-	0.08	-	0.13	-	0.01	-	0.04	-
**Reference H5N1**^c^	2.02	✓✓✓	-0.05	-	-0.01	-	-0.03	-	0.03	-	0.03	-	0.00	-	0.00	-	0.02	-	0.01	-	0.01	-	0.04	-
**1N5**^d^	2.63	✓✓✓	0.01	-	0.02	-	0.01	-	0.00	-	0.01	-	0.02	-	0.03	-	0.03	-	0.01	-	0.00	-	0.00	-
**2D16**^d^	3.30	✓✓✓	0.02	-	0.01	-	0.01	-	0.00	-	0.02	-	0.03	-	0.01	-	0.03	-	0.00	-	-0.01	-	-0.01	-
**2E14**^d^	2.62	✓✓✓	0.35	✓	0.13	-	0.02	-	0.00	-	0.00	-	0.10	-	0.54	✓✓	0.01	-	0.01	-	0.00	-	0.00	-
**2G14**^d^	2.26	✓✓✓	0.01	-	0.00	-	0.05	-	0.00	-	0.00	-	0.00	-	0.01	-	0.01	-	0.03	-	0.00	-	0.03	-
**3D23**^d^	1.69	✓✓✓	0.04	-	0.02	-	0.09	-	0.02	-	0.02	-	0.03	-	0.02	-	0.08	-	0.06	-	0.01	-	0.01	-
**3H4**^d^	2.58	✓✓✓	0.01	-	0.01	-	0.02	-	0.00	-	0.01	-	0.02	-	0.01	-	0.02	-	0.03	-	0.00	-	-0.01	-
**Rab-1**^e^	1.86	✓✓✓	-0.10	-	0.08	-	1.82	✓✓✓	-0.13	-	-0.19	-	-0.14	-	-0.13	-	0.13	-	1.46	✓✓✓	1.31	✓✓✓	-0.04	-
**Rab-2**^e^	0.49	✓	-0.48	-	-0.34	-	0.38	✓	-0.53	-	-0.53	-	-0.52	-	-0.52	-	-0.45	-	0.42	✓	0.24	✓	-0.40	-
**Rab-3**^e^	1.64	✓✓✓	-0.16	-	0.01	-	1.46	✓✓✓	-0.25	-	-0.25	-	-0.23	-	-0.25	-	-0.04	-	1.27	✓✓✓	1.13	✓✓✓	-0.10	-
**Rab-4**^e^	0.68	✓✓	-0.43	-	-0.28	-	0.55	✓✓	-0.46	-	-0.46	-	-0.45	-	-0.45	-	-0.35	-	0.60	✓✓	0.39	✓	-0.34	-
**Rab-5**^e^	1.26	✓✓✓	-0.20	-	-0.04	-	1.45	✓✓✓	-0.22	-	-0.22	-	-0.20	-	-0.20	-	-0.02	-	1.14	✓✓✓	1.01	✓✓✓	-0.14	-
**Rab-6**^e^	1.41	✓✓✓	-0.26	-	-0.01	-	1.76	✓✓✓	-0.30	-	-0.30	-	-0.28	-	-0.28	-	-0.13	-	0.94	✓✓	1.44	✓✓✓	-0.12	-
**Rab-7**^e^	1.46	✓✓✓	-0.27	-	-0.03	-	1.25	✓✓✓	-0.31	-	-0.30	-	-0.29	-	-0.29	-	-0.14	-	1.34	✓✓✓	1.01	✓✓✓	-0.13	-
**Rab-8**^e^	0.68	✓✓	-0.43	-	-0.28	-	0.55	✓✓	-0.46	-	-0.46	-	-0.45	-	-0.45	-	-0.35	-	0.60	✓✓	0.39	✓	-0.34	-

For statistical analysis, please refer to S1 File.

^a^Chickens exposed to A/Ck/West Java/Sbg-29/2007

^b^Chickens exposed to A/Ck/West Java/Sbg-29/2007

^c^Chickens exposed to A/chick/Scotland/59

^d^Mice immunised with KLH-M2e_2-19_

^e^Rabbits immunised with KLH-M2e_2-19_

### Chicken sera recognized at least 2 different epitopes spanning M2e residue 5–18 and 10–17

**Fig 1:**
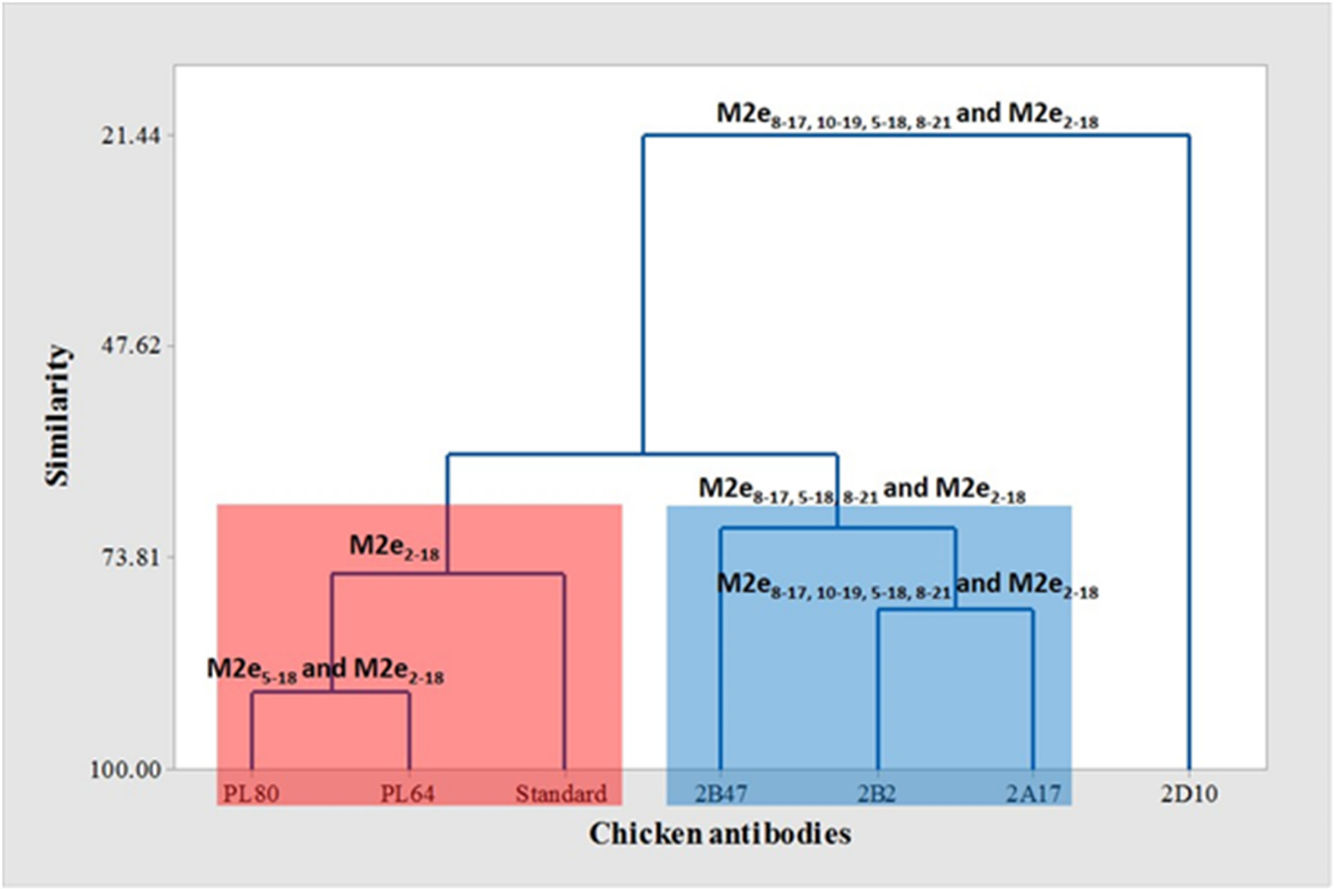
Clustering based on average linkage algorithm illustrates the similarity of chicken antibodies reactivity to the M2e peptides as indicated on the nodes of each group. Left to right: Cluster 1 (red box) chicken sera which reacted with M2e_5-18_ and M2e_2-18_; Cluster 2 (blue box) chicken sera which reacted with M2e_8-17, 10–19, 5–18, 8–21_ and M2e_2-18_; and 2D10 chicken serum which reacted with M2e_8-17, 10–19, 5–18, 8–21_ and M2e_2-18_.

M2e mapping ELISA results revealed a distinctive reactivity pattern between the chicken sera exposed to the A/Ck/West Java/Sbg-29/2007 (Sbg-29/2007) (*n* = 4) and A/Ck/West Java/PWT-WIJ/2006 (PWT/2006) (*n* = 2). Anti-M2e sera from chickens exposed to Sbg-29/2007 (2A17, 2B2, 2B47 and 2D10) showed a range of medium to strong reactivity to M2e_8-21_, strong reactivity to M2e_5-18_ and weak to strong reactivity to M2e_8-17_ (Table 3). With the exception of cAb 2B47, Sbg-29/2007 antisera also showed a range of weak to strong reactivity to M2e_10-19_. Non-reactivity of cAb 2B47 to M2e_10-19_ was not fully understood, but this particular cAb was only reactive to peptides which included residues E8 and T9 (Fig 1). Collectively, Sbg-29/2007 antisera showed reactivity to peptides which shared a minimum of eight residues (^10^PTRNEWEC^17^) of the M2e (Fig 1).

While Sbg-29/2007 antisera were reactive to peptides with 10 residues (M2e_8-17_ and M2e_10-19_), as well as 14 residues (M2e_5-18_ and M2e_8-21_), chicken antisera to PWT/2006 (PL64 and PL80) were only reactive to the 14 residues M2e_5-18_ (Table 3). Despite M2e_5-18_ sharing residues with the whole M2e_6-15_ and M2e_8-17_, and most residues in M2e_4-13_ and M2e_10-19_, neither of the PWT/2006 antisera reacted to any of these shorter peptides. This suggested that these 10-residue peptides were inadequate to represent the PWT/2006-strain epitope which elicited antibody responses in the chickens.

**Fig 2:**
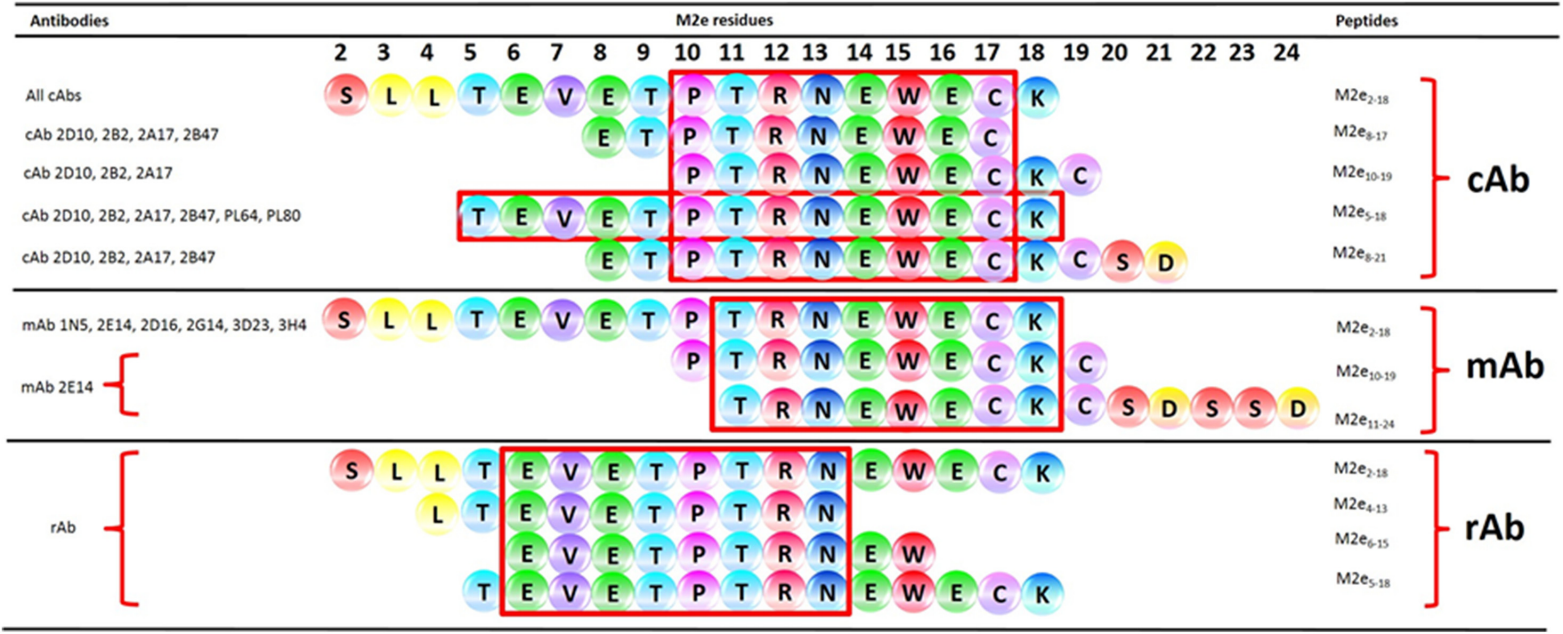
The antigenic determinants of M2e protein recognized by chicken, mouse and rabbit antibodies highlighted with the red boxes. In the order from top to bottom, chicken antibodies to Sbg-29/2007 strain that recognized peptides containing residues ^10^PTRNEWEC^17^; chicken antibodies to PWT/2006 strain recognized peptides with residues ^5^TEVETPTRNEWECK^18^; mouse monoclonal antibodies recognized peptides with residues ^11^TRNEWECK^18^ and rabbit antibodies recognized peptides with residues ^6^EVETPTRN^13^. Tested antibodies were listed on the left, while the peptides corresponding to the residues recognized by each group are indicated on the right.

Although the reference H5N1 serum (produced against A/chick/Scotland/59 strain) was commercially generated based on its hemagglutinin inhibition titer, it showed strong reactivity to peptide M2e_2-18_ (mean OD_450_ 2.02) (Table 3). However, no reactivity was observed between the reference sera and any of the mapping peptides. Alignment of the peptides recognized by the chicken sera showed that at least two epitopes, in addition to the immunogen, were recognized, namely M2e_5-18_ (^5^TEVETPTRNEWECK^18^) and M2e_10-18_ (^10^PTRNEWECK^18^) (Table 3, Fig 2). Both epitopes contained residues M2e 10–17, which are recognised by all cAbs and which correspond to the most hydrophilic part of the M2e protein (residues 12 to 20) (Fig 2).

### Chicken sera reactivity pattern is highly influenced by its immunogen as well as individual chicken immune response

**Fig 3:**
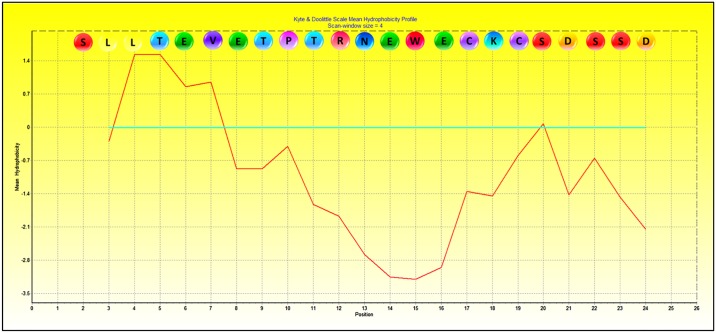
Hydrophobicity plot of M2e protein sequence (residue 2 to 24) based on Kyte & Doolittle scale mean of hydrophobicity profile in BioEdit.

Clustering analysis of chicken antisera based on their reactivity with M2e peptides revealed two major clusters broadly related to the antigen used to immunise the donor chickens (Fig 3). Cluster 1 grouped Sbg-29/2007 antisera together, particularly 2B2, 2A17 and 2B47, based on their reactivity to M2e_8-17, 10–19, 5–18, 8–21_ and M2e_2-18_; while cluster 2 grouped PWT/2006 antisera (PL64 and PL80), based on their reactivity to M2e_5-18_ and M2e_2-18_, along with the reference H5N1 sera (produced against A/chick/Scotland/59) which only reacted to peptide M2e_2-18_.

Although cAb2D produced against the Sbg-29/2007 strain shared a similar reactivity pattern with cAbs 2B2 and 2A17 (M2e_8-17, 10–19, 5–18, 8–21_ and M2e_2-18_), clustering analysis recognized cAb 2D10 sera as the least similar to the other sera. Observation of its OD_450_ readings showed that cAb 2D10 reacted strongly with all five peptides (OD_450_ 2.02–2.33) (Table 3) which was not observed with the other sera. And uniquely this sera also had high anti-M2e antibodies titre (1:10,240).

### Mouse monoclonal antibodies recognized epitopes M2e_2-18_ and M2e_11-18_ while rabbit polyclonal antibodies recognized epitope M2e_6-13_

M2e comparative mapping by mAbs showed minimal variability in their reactivity patterns. While all six mAbs strongly reacted with peptide M2e_2-18_ (OD_450_ 1.69–3.30), only mAb 2E14 showed a weak and medium reactivity to M2e_10-19_ and M2e_11-24_, respectively (Table 3). Together, mAbs recognized an M2e epitope containing a minimum of eight residues (^11^TRNEWECK^18^) to 17 residues (^2^SLLTEVETPTRNEWECK^18^), in which the epitopes mostly overlapped with the epitope recognized by cAbs described above (Fig 2).

Apart from the similar strong reactivity observed for peptide M2e_2-18_ (OD_450_ 1.73), rAbs also demonstrated strong reactivity to M2e_4-13,_ M2e_6-15_ and M2e_5-18_ (Table 3), a combination which was not demonstrated in the previous two groups of antibodies. All these peptides shared residues ^6^EVETPTRN^13^ which indicated that the epitope recognized by rabbit was different from the chicken and mouse antibodies.

Comparison of the M2e epitopes recognized for all three groups of antibodies clearly showed that the chicken, mouse and rabbit sera recognized five epitopes, namely M2e residues 2–18 for all antibodies, with specifically M2e residues 5–18 and 10–17 recognized by the cAbs, M2e residues 11–18 recognized by one mAb, and M2e residues 6–13 by the rAbs (Fig 2). The shorter epitopes represented by the different antibodies group was recognized on two different sites of the M2e protein. cAbs and mAbs antibodies recognized epitopes located at the central region of the M2e protein (^10^PTRNEWECK^18^), while the rAb antibodies recognized an epitope located at the N-terminal of the M2e protein (^6^EVETPTRN^13^) (Fig 2).

## Discussion

Based on our previous success in demonstrating the effective use of M2e protein as a target for DIVA strategy, we attempted to develop a competitive ELISA test targeting the M2e protein. This test was anticipated to possess a broad host species applicability which is capable of DIVA for a simple yet effective AIV surveillance tool in domestic poultry. We have here described the comparative mapping of anti-M2e antibodies from chickens, mice and rabbits. Our findings revealed the occurrence of two separate epitopes on the M2e protein, where one epitope was exclusively recognized by the rAbs antibodies, while the other was recognized by both mAb and cAbs. It is important to note that for development of a competitive ELISA, the test and competitor antibodies need to cross-react with the same, or at least similar epitope, within the same antigen. Such is the case where cAbs are the test antibodies, while mAbs but not rAbs are the potential competitors.

Despite the difference in the immunogen used for anti-M2e antibody production in mice and rabbits (KLH-conjugated peptide) versus chickens (H5N1 live virus), our findings that cAbs, mAbs and rAbs recognized five M2e epitopes within the sequence ^2^SLLTEVETPTRNEWECK^18^ was similar to those of others [13, 14, 20, 34, 35, 39–45] (Table 4). The high frequency of epitope ^6^EVETPTRN^13^ occurrence in the previous studies suggests that it is likely to be a dominant epitope for M2e protein. Additionally, epitope ^6^EVETPTRN^13^ is potentially a major epitope for rAbs, whereas a previous study on immunization of rabbits and mice using M2e_2-10_^2^SLLTEVETP^10^ conjugated with KLH (SP1-KLH) showed to be more immunogenic in rabbits than it was in mice [40].

**Table 4:** Summary of epitopes recognized on influenza A virus M2e protein by different antibodies.

Antibody type and designation	Antibody source	Immunogen	Epitope sequence (Identifying Antibody)	Residue length	References
Polyclonal (AS1, AS2, AS3, AS4)	Rabbit	Fusion-M2e (BSA)	^2^SLLTEVETPIR^12^	11	[13]
Monoclonal (8C6, 1B12)	Mice	Fusion-M2e (GST)	^6^EVETPIRN^13^ ^2^SLLTEVETPIRNEW^15^	8 14	[39, 44, 45]
Monoclonal	Mice	Live virus & synthetic peptide	^4^LTEVETPIRNEWG^16^	13	[43]
Monoclonal (L66, N547, Z3G1, C40G1, 14C2)	Human (λ HAC or KM^™^ mice)	Fusion-M2e (BSA)	^2^SLLTEVETPIRNEWG^16^ (L66) ^3^LLTEVETPIRNEWG^16^ (N547) ^3^LLTEVETPIR^12^ (Z3G1)^9^TPIRNE^14^ (C40G1) ^6^EVETPIRNEW^15^ (14C2)	15 14 10 6 10	[14, 42]
Monoclonal	Mice	Fusion-M2e (BSA)	^2^SLLTEVET^9^ (M2e8-7) ^3^LLTEVETPIR^12^ (Z3G1)	8 10	[34]
Monoclonal	Mice	Fusion-M2e (BSA)	^4^LTEVETPIRN^12^ (L18) ^2^SLLTEVET^9^ (O19) ^2^SLLTEVETPIRNEWGCRNDSSD^24^ (P6) ^7^VETPIRN^13^ (S1)	108 23 7	[41]
Polyclonal	Mice		^2^SLLTEVETPIRNEWG^16^	15	[20]
Monoclonal	Human		^2^SLLTE^6^ (TCN-031, TCN-032)	5	[35]
	Mice	Fusion-M2e (KLH)	^2^SLLTEVETP^10^	9	[40]
Polyclonal & monoclonal	Chicken, mice, rabbit	Live virus & fusion-M2e (KLH)	^5^TEVETPTRNEWECK^18^ (cAbs) ^10^PTRNEWEC^17^ (cAbs) ^2^SLLTEVETPTRNEWECK^18^ (cAbs, mAbs, rAbs)^11^TRNEWECK^18^ (mAb) ^6^EVETPTRN^13^ (rAbs)	14 8 17 8 8	This study

Difference at residue I11T between the current and previous studies corresponded to the human and swine specific M2e sequence in the former (I11) and avian specific M2e sequence in the latter (T11) [57].

Minimal variation observed for mAbs was likely due to the double selection using M2e_2-19_-KLH-based ELISA for hybridoma production and final selection. This limited the mAbs reactivity only to the immunogen with low cross reactivity to the other peptides used in the study. Nevertheless, one mAb recognized two other peptides which contain residues M2e 11–18 (Fig 2) that overlapped with M2e epitopes recognized for cAbs. Hence, mAb was suggested to be a better competitor in a cELISA-based test for cAbs in contrast to rAbs, as the latter showed fewer overlapping residues (Fig 2).

However, it was notable that one mAb and the majority of cAbs showed slight variation in peptide recognition. Although the antigenic determinants recognized by the mAb and cAbs in the current study overlapped with the epitopes found previously (residues 5 to 16 of M2e) [14, 20, 42–44], they differed in that two of the recognized epitopes (^10^PTRNEWEC^17^ for cAbs, ^11^TRNEWECK^18^ for mAb) extended further from the mid-region into the C-terminal end of the M2e protein (Table 4). Both were shorter epitopes (8 aa in length) and independent of the N-terminal peptide (M2e_2-9_), with one or two more residues at the epitope C-terminal (C17 and K18) than previously reported epitopes recognized in humans and mice. This suggests that residues ^2^SLL^4^ is a less important antigenic determinant in chickens and rabbits than it is in humans [35]. Conversely, C17 and K18 may possibly be important residues for cAbs epitope recognition. Importance of K18 for mAb epitope recognition was also suggested by the reported loss of anti-M2e antibody responses following immunization with truncated M2e_2-16_ in a vaccine study in mice [20]. Difference by two to three residues between the M2e epitopes recognized by mAbs has also been described previously [43]. Zhang et al. (2006) suggested that such observations could be due to either a true existence of species-related variation in epitope recognition, or difference in assay sensitivity used for epitope recognition, or both [43]. Epitope variation was observed in a separate M2e-unrelated study in rabbits using 10 human proteins, where although the epitopes recognized for a single protein were similar, they were not identical [46]. The epitopes recognized by mAbs in the current study represent another species-related variation of the existing recognized M2e epitopes, while this is the first known M2e epitope reported in chickens. Nevertheless, M2e residue C17 and K18 may be of contributing to the antigenic characteristics of M2e.

M2e protein residues S2, T5, E6, P10, I11, E14 and W15 have been identified as critical for antibody interactions [34, 35, 44, 47–49]. Epitope studies have suggested that charged residues (E, K an D), and polar residues (R, N, Q, P and T) are preferred in highly antigenic epitopes [50, 51], where the hydrophilic amino acids (R, K, N, P, H, D and E) are more prominent [52]. A recent analysis of the M2e crystal structure complexed with monoclonal antibody has recognized that residues T5, E6, V7, P10, R12 and N13 assist M2e hydrophilic interactions, which contributes to epitope accessibility in antigen-antibody binding [47]. Amino acid variation at residues P10, E14 and E16 resulted in predicted M2e structural differences between two H5N1 strains, Vietnam/1194/04 and Hong Kong/156/97 [53]. The latter H5N1 strain showed a folded hairpin structure that limits antigen recognition in comparison to a relatively more accessible structure observed in the former. M2e protein sequence is not available for PWT/2006 strain used in current study. The M2e amino acid sequence of A/chick/Scotland/59 (EMBL accession number CY015082) and A/Ck/West Java/Sbg-29/2007 (H5N1) (GenBank accession number AKI82362.1) only differs by residue E14G for Scotland/59, and K18C for both from the M2e A/Vietnam/1194/04, hence a similar ‘open’ structure is likely for the Sbg-29/2007 M2e protein.

It is noted that antibodies from chickens exposed to two different strains of H5N1 in current study recognized two dominant but overlapping epitopes on the M2e protein. Differences observed may be related to the M2e membrane-bound protein conformation of these two H5N1 strains. Factors such as degree of protein protrusion from membrane surface [54], as well as its accessibility for binding activities [55] highly influence the whole presentation of the protein to the birds immune system. Reactivity with only the 14 aa mapping peptide (M2e_5-18_, ^5^TEVETPTRNEWECK^18^) observed for sera PWT/2006 may be related to the structural element formed by the protein on the virus particle. Previous study on the human tryptophanyl-tRNA synthetase epitopes using 10 aa and 15 aa peptides has demonstrated similar observations [56]. It was suggested that the 10 aa peptides (M2e_4-13, 6–15, 8–17 and 10–19_) were not sufficient to imitate the functional structure of the epitope since it is located in a loop structure partially characterized by an α-helix. In the case of the M2e protein, its three-dimensional structure showed a compact U-shaped conformation, where a β-turn structure is adopted by residues T5 to E8, and 3_10_ helix from residues I11 to W15 [47]. Hence, it was likely that although the two epitopes residues overlap, the PWT/2006 sera were only reactive to the 14 aa peptide M2e_5-18_ due to the lack of complete residue for a functional epitope formed by the 10 aa peptides.

Difference in length of recognized epitopes in anti-M2e cAbs may be related to the different degree of virus virulence between the H5N1 strains and individual chicken immune responses. Strong reactivity to the M2e peptides observed for the 2D chick sera in current study was reasoned to be due to the double boosts vaccination using killed virus, followed by a live virus challenge. Current findings revealed that the Sbg-29/2007 antisera were capable of recognising shorter epitopes in comparison to the PWT/2006 strain. Slight differences in signal intensity for each identified peptide for Sbg-29/2007 antisera were also noted in relation to the number of vaccinations for each individual birds. Previous study on epitope patterns in rabbit’s parallel immunizations with a single antigen showed that polyclonal response in individual animal may differs in their affinities [46]. Also, the difference in the immunogen used was implicated in the lack of response to the mapping peptides observed for the reference H5N1 sera. Temporal and spatial distant origin of the strain used for immunisation (Scotland/59) from the strain used as the basis for the mapping peptide design (PWT-WIJ//2006) has likely influenced this particular cAb reactivity.

Although the relatively limited number of serum samples available for testing in the current study do not represent the complexity of antibody response to M2e protein, nevertheless, the results presented provided information on differences of Me2 epitope recognition by mouse, rabbit and chicken antibodies. Identification of antigenic determinants or epitopes of the target protein will enable us to formulate the most suitable source of anti-M2e antibodies for further development.

In summary, we have identified five epitopes spanning residue 2 to 18 of M2e protein for mouse, chicken and rabbit sera with variations in length (8 to 17 aa) from two localities on the M2e protein (N-terminal and mid-region). We also concluded that mouse anti-M2e antibodies are more suitable to be used as a competitor antibodies than the rabbit anti-M2e antibodies for further work on M2e-based competitive ELISA diagnostic test. This was highly suggestive by the overlapping epitopes (^11^TRNEWEC^17^) demonstrated by both chicken antibodies and one of the mouse antibodies.

## Supporting Information

S1 File Detailed statistical analysis performed in this study. (DOCX)
