# Supplementary material for: Epitope Mapping of Avian Influenza M2e Protein: Different Species Recognise Various Epitopes
Source: PLoS One. 2016 Jun 30;11(6):e0156418. doi: 10.1371/journal.pone.0156418 (PMC4928777; doi:10.1371/journal.pone.0156418)
Supplement: S1 File — (DOCX) [file pone.0156418.s001.docx]

**Supplementary 1.** Detailed statistical analysis performed in this study.

**Supplementary Table 1.** Comparisons of mean OD_450_ readings for chicken antibodies reactivity to the M2e peptides (antigen). Overall mean, significance value and R-squared value (R^2^) of antigen reactivity against different antibodies are listed horizontally (bottom row), while the values for antibody reactivity against different antigen are listed vertically (last 3 columns). Mean OD_450_ readings that do not share a letter are significantly different at p=0.05 according to Tukey Pairwise Comparisons.

|  | **Antigen** | | | | | | | | | | | | | | | | | | | | | | | |  |  |  |
| --- | --- | --- | --- | --- | --- | --- | --- | --- | --- | --- | --- | --- | --- | --- | --- | --- | --- | --- | --- | --- | --- | --- | --- | --- | --- | --- | --- |
| **Antibody** | **17-mer** | | **11** | | **10** | | **9** | | **8** | | **7** | | **6** | | **5** | | **4** | | **3** | | **2** | | **1** | | **Mean** | **ANOVA** | **R^2^** |
| **2D10** | 2.33 | A | 0.04 | AB | 2.14 | A | 2.14 | A | 0.10 | AB | 0.10 | A | 0.14 | A | 2.02 | A | 2.24 | A | 0.17 | AB | 0.09 | A | 0.05 | AB | 0.96 | p=0.01 | 99.97% |
| **PL64** | 2.29 | AB | 0.04 | AB | 0.01 | E | 0.76 | F | 0.08 | BC | 0.08 | AB | 0.14 | A | 0.02 | D | 0.10 | E | 0.10 | AB | 0.07 | AB | 0.10 | A | 0.32 | p=0.01 | 99.82% |
| **PL80** | 2.34 | A | 0.10 | A | -0.05 | E | 1.13 | E | 0.16 | A | 0.02 | C | 0.07 | B | -0.02 | D | 0.08 | E | 0.13 | AB | 0.01 | C | 0.04 | AB | 0.33 | p=0.01 | 99.60% |
| **2A17** | 2.11 | BC | 0.02 | AB | 0.92 | C | 1.66 | B | 0.04 | BC | 0.05 | BC | 0.04 | BC | 0.81 | B | 0.87 | C | 0.17 | AB | 0.03 | BC | 0.06 | AB | 0.56 | p=0.01 | 99.03% |
| **2B2** | 2.07 | C | 0.01 | AB | 0.58 | D | 1.51 | C | 0.02 | C | 0.04 | BC | 0.05 | B | 0.26 | C | 0.35 | D | 0.20 | A | -0.01 | C | -0.01 | B | 0.42 | p=0.01 | 99.08% |
| **Reference H5N1** | 2.02 | CD | -0.05 | B | -0.01 | E | -0.03 | G | 0.03 | BC | 0.03 | C | 0.00 | C | 0.00 | D | 0.02 | E | 0.01 | BC | 0.01 | C | 0.04 | AB | 0.17 | p=0.01 | 99.83% |
| **2B47** | 1.85 | D | -0.22 | C | 1.20 | B | 1.32 | D | -0.08 | D | -0.27 | D | -0.12 | D | -0.20 | E | 1.13 | B | -0.11 | C | -0.21 | D | -0.13 | C | 0.34 | p=0.01 | 99.91% |
| **Mean** | 2.14 |  | -0.01 |  | 0.68 |  | 1.21 |  | 0.05 |  | 0.01 |  | 0.05 |  | 0.41 |  | 0.68 |  | 0.10 |  | 0.00 |  | 0.02 |  |  |  |  |
| **ANOVA** | p=0.01 | | p=0.01 | | p=0.01 | | p=0.01 | | p=0.01 | | p=0.01 | | p=0.01 | | p=0.01 | | p=0.01 | | p=0.01 | | p=0.01 | | P=0.01 | |  |  |  |
| **R^2^** | 84.61% | | 84.78% | | 98.83% | | 99.28% | | 86.79% | | 98.17% | | 99.96% | | 99.65% | | 99.57% | | 66.69% | | 95.67% | | 72.86% | |  |  |  |

**Supplementary Table 2.** Comparisons of mean OD_450_ readings for mouse antibodies reactivity to the M2e peptides (antigen). Overall mean, significance value and R-squared value (R^2^) of antigen reactivity against different antibodies are listed horizontally (bottom row), while the values for antibody reactivity against different antigen are listed vertically (last 3 columns). Mean OD_450_ readings that do not share a letter are significantly different at p=0.05 according to Tukey Pairwise Comparisons.

|  | **Antigen** | | | | | | | | | | | | | | | | | | | | | | | |  |  |  |
| --- | --- | --- | --- | --- | --- | --- | --- | --- | --- | --- | --- | --- | --- | --- | --- | --- | --- | --- | --- | --- | --- | --- | --- | --- | --- | --- | --- |
| **Antibody** | **17-mer** | | **11** | | **10** | | **9** | | **8** | | **7** | | **6** | | **5** | | **4** | | **3** | | **2** | | **1** | | **Mean** | **ANOVA** | **R^2^** |
| **1N5** | 2.63 | AB | 0.01 | B | 0.02 | A | 0.01 | A | 0.00 | A | 0.01 | A | 0.02 | BC | 0.03 | B | 0.03 | B | 0.01 | B | 0.00 | A | 0.00 | A | 0.23 | p=0.01 | 99.30% |
| **2D16** | 3.30 | A | 0.02 | B | 0.01 | A | 0.01 | A | 0.00 | A | 0.02 | A | 0.03 | B | 0.01 | B | 0.03 | B | 0.00 | B | -0.01 | A | -0.01 | A | 0.28 | p=0.01 | 83.69% |
| **2E14** | 2.62 | AB | 0.35 | A | 0.13 | A | 0.02 | A | 0.00 | A | 0.00 | A | 0.10 | A | 0.54 | A | 0.01 | B | 0.01 | B | 0.00 | A | 0.00 | A | 0.32 | p=0.01 | 99.70% |
| **2G14** | 2.26 | AB | 0.01 | B | 0.00 | A | 0.05 | A | 0.00 | A | 0.00 | A | 0.00 | C | 0.01 | B | 0.01 | B | 0.03 | AB | 0.00 | A | 0.03 | A | 0.20 | p=0.01 | 99.09% |
| **3D23** | 1.69 | B | 0.04 | B | 0.02 | A | 0.09 | A | 0.02 | A | 0.02 | A | 0.03 | B | 0.02 | B | 0.08 | A | 0.06 | A | 0.01 | A | 0.01 | A | 0.17 | p=0.01 | 53.13% |
| **3H4** | 2.58 | AB | 0.01 | B | 0.01 | A | 0.02 | A | 0.00 | A | 0.01 | A | 0.02 | BC | 0.01 | B | 0.02 | B | 0.03 | AB | 0.00 | A | -0.01 | A | 0.23 | p=0.01 | 99.98% |
| **Mean** | 2.51 |  | 0.07 |  | 0.03 |  | 0.03 |  | 0.00 |  | 0.01 |  | 0.03 |  | 0.10 |  | 0.02 |  | 0.02 |  | 0.00 |  | 0.00 |  |  |  |  |
| **ANOVA** | p=0.05 | | p=0.01 | | Not significant | | Not significant | | Not significant | | Not significant | | p=0.01 | | p=0.01 | | p=0.01 | | p=0.01 | | Not significant | | Not significant | |  |  |  |
| **R^2^** | 19.96% | | 93.95% | | 0.0% | | 0.0% | | 0.0% | | 0.0% | | 91.78 | | 97.28% | | 82.95% | | 64.1% | | 0.0% | | 0.0% | |  |  |  |

**Supplementary Table 3.** Comparison of OD_450_ reading for rabbit antibody reactivity to the M2e peptides (antigen). Mean OD_450_ readings that do not share a letter are significantly different at p=0.05 according to Tukey Pairwise Comparisons.

| **Antigen** | **Rabbit antibody** | |
| --- | --- | --- |
| 17-mer | 1.73 | B |
| 11 | -0.07 | F |
| 10 | 0.12 | E |
| 9 | 1.96 | A |
| 8 | -0.10 | F |
| 7 | -0.10 | F |
| 6 | -0.07 | F |
| 5 | -0.08 | F |
| 4 | 0.14 | E |
| 3 | 1.58 | C |
| 2 | 1.42 | D |
| 1 | 0.00 | F |
| **ANOVA** | p=0.01 |  |
| **R^2^** | 99.74% | |
